# Effects of Friction Stir Welding on the Mechanical Behaviors of Extrusion-Based Additive Manufactured Polymer Parts

**DOI:** 10.3390/polym15153288

**Published:** 2023-08-03

**Authors:** Jin-Feng Liu, Ying-Guo Zhou, Shu-Jin Chen, Shao-Qiang Ren, Jun Zou

**Affiliations:** 1 School of Materials Science and Engineering, Jiangsu University of Science and Technology, Zhenjiang 212003, China; 2 Aromatics Department, Zhejiang Petrochemical Co., Ltd., Zhoushan 316021, China

**Keywords:** friction stir welding (FSW), extrusion-based additive manufacturing (EAM), PETG

## Abstract

The friction stir welding (FSW) of thermoplastic polymers is gradually receiving attention because of its advantages including high efficiency and pollution-free manufacturing. The extrusion-based additive manufacturing (EAM) of polymers has also become one of the main processing methods for thermoplastic parts. In this paper, a hybrid manufacturing method for the FSW process and EAM technology is proposed and explored. The effects of the FSW process using two different welding tools on the mechanical behaviors of 3D printing polymer parts were compared and investigated and the corresponding mechanism was analyzed. The results show that the appropriate welding tool is beneficial for eliminating the anisotropy and decreasing the porosity of 3D-printed parts. Therefore, the improving effects of the FSW process on the mechanical behaviors of the EAM parts are verified. The mechanism was attributed to the high-speed rotation of the welding tool with the appropriate shape, which can promote the flow of polymer melt in the welding region, leading to the formation of dense structures caused by the entanglement of the molecular chains. This study may provide some assistance in modern industrial manufacturing for the processing of large custom components.

## 1. Introduction

With the development of industry and the requirement for lightweight materials, thermoplastic polymers have been widely used in various fields because of their excellent physical properties such as high strength, light weight, and easy processing [1,2,3,4,5]. Polymer joining is becoming an increasingly important research topic [6,7,8]. However, compared to widely used metal joining technology [9,10,11], research on polymer joining is relatively scarce. In the early stages, mechanical connection and bonding are two main methods of polymer joining. However, both methods have their corresponding defects. For example, mechanical connection generally uses screws and/or other fasteners to achieve the combination of parts, and its essence is a mechanical interlocking mechanism. It will definitely increase the weight of the materials and is not beneficial to the appearance of the resulting products. As for the bonding process, the adhesive strength, which depends on the strength of the adhesive and the interface layer, remains the main limiting factor for its wider application. Zhang et al. fabricated a super adhesive, and the bonding strength was reported to be as high as 10.2 MPa [12]. However, this value is still much lower than the self-strength of the thermoplastic polymer used. As a result, the bonding process is suitable only for some low-strength working environments.

Welding has gradually become one of the main methods of thermoplastic polymer connection. Similar to what occurs in metal welding, the main means of polymer welding include heating fusion welding, pressure welding, indirect welding, etc. Heating fusion welding is relatively widely used among the three types of methods, and it can be further classified into laser welding, ultrasonic welding, resistance heating welding, etc. Representatively, Femandes investigated the laser welding technology of thermoplastic polymers [13] and Volkov studied the ultrasonic welding of rigid plastics with additional compressive force [14]. Resistance heating welding has been realized to require heating sources and the working time is long, laser welding equipment is expensive and requires high surface quality of parts, and ultrasonic welding process has a limited power and requires a special workpiece shape. There are basically no reports on the welding of polymers using the third type of welding.

The degree of research interest and application of polymer pressure welding are balanced between heating fusion welding and indirect welding. Friction stir welding (FSW) is a typical representative of pressure welding that uses a welding tool to generate heat and plasticize workpieces via high-speed rotation. The application of FSW technology in the field of thermoplastics began in 1997. However, it was only after 2009 that thermoplastic polymer FSW was really explored in a deeper way. In general, the thermoplastic polymers that are the most concerned are polyethylene (PE) and polypropylene (PP) [15,16,17]. Furthermore, acrylonitrile butadiene styrene terpolymer (ABS), polyvinyl chloride (PVC), nylon (PA), polytetrafluoroethylene (PTFE), polymethyl methacrylate (PMMA), polyethylene terephthalate (PET), and carbon fiber reinforced polyether ether ketone (CF-PEEK) have also been investigated [18,19,20,21,22,23]. Compared to other welding technologies, the FSW of thermoplastic polymers has great advantages in cost, efficiency, and green pollution-free manufacturing. Furthermore, because the mechanical properties of thermoplastic polymer welded joints depend mainly on the molecular entanglement density in the corresponding welded region, the high-speed rotation of the welding tool will drive the polymer chain to move along with the rotational direction during the FSW process, thus improving the flow of polymers, resulting in increased welding strength.

Polymer extrusion-based additive manufacturing (EAM), one of the fastest growing additive manufacturing technologies (AM, which is also commonly known as three-dimensional printing, or 3D printing), has gradually become a major thermoplastic part processing method [24,25,26,27,28]. In a typical EAM process, the heated and melted filament of thermoplastics is extruded from a nozzle and then deposited on a printing bed accordingly, and hence, a complex 3D component is produced. Therefore, to some extent, EAM can be seen as a continuous welding process of heated material. Over the past decade, EAM technology has been widely studied and two types of common defects have been well recognized. Firstly, it is inevitable for pores to form between the deposited polymer caused by the shrinkage of the polymer melts, resulting in weak interfacial adhesion [29]. Second, it is obviously easy for EAM technology to cause anisotropy [30]. In order to improve the applicability of 3D-printed parts, a combined method of the EAM technology with other manufacturing processes can be easily thought of. The FSW process is such an attempt.

In this paper, a hybrid manufacturing method of the FSW process and EAM technology is proposed and explored. To enhance the mechanical properties of the printing parts, a new welding tool is designed and used according to the anisotropy and internal pore characteristics of the 3D printing parts. The influence of the welding tool on the quality of the FSW joints of 3D printing plastic parts is investigated and then the corresponding mechanism is analyzed. It was found that the appropriate welding tool is beneficial to the welding of 3D-printed parts; in particular, it can effectively reduce the anisotropy of 3D-printed parts. Furthermore, it was realized that FSW using the right tool can reduce the number of internal pores in printed parts and form a relatively tight interface, which is attributed to the high-speed rotation of the welding tool, suggesting that the dynamical flow of the polymer melt in the welding region can promote entanglement between the molecular chains.

## 2. Experiment

### 2.1. Materials and Equipment

In this study, Poly(ethylene terephthalateco-1,4-cylclohexylenedimethylene terephthalate) (PETG) was selected as the main raw material due to its excellent easy-processing ability, near-perfect transparency, excellent mechanical properties such as a comprehensive balance of strength, stiffness, and toughness, and very wide service temperature range [28,31]. The PETG used was purchased from Huarun Chemical Co., Ltd., Changzhou, China and its melt flow volume was about 6.1 cm^3^/10 min (230 °C, 38 N).

The experimental welding equipment used is a self-made double shoulder FSW machine, and the maximum welding speed of the machine can be up to 20,000 r/min. In the welding process, an inclination angle of 1–3° was used between the welding tool and the workpiece table, which can effectively reduce the pressure of the welding tool and protect the pin from breaking. The EAM machine used is a self-made four-axis 3D printer (the precision can be as high as 0.05 mm). In addition, a twin-screw extruder (SHJ30, Lianjiang, Zhangjiagang, China) was used to fabricate the PETG filament.

### 2.2. Experimental Process

**Figure 1:**
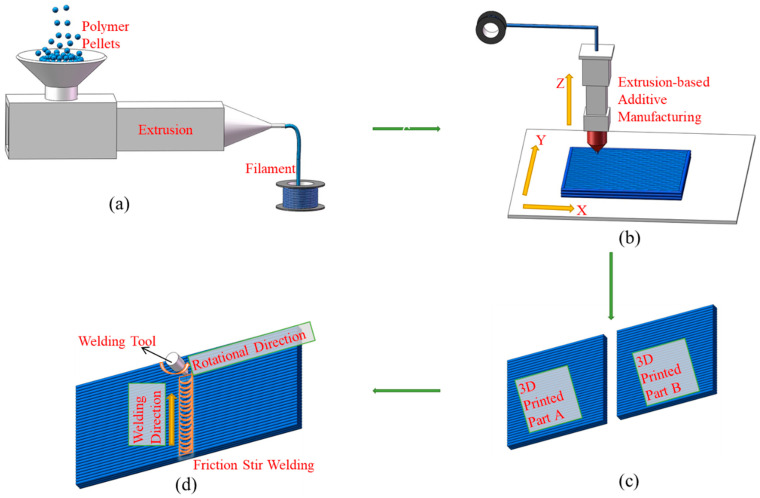
Schematic diagram of the FSW process for 3D-printed parts, which includes: (**a**) filament fabrication, (**b**) 3D printing, (**c**) a welding workpiece, and (**d**) the FSW process.

Figure 1 shows the specific experimental schematic diagram. It can be divided into the following four subpictures, that is, subpictures showing (a) filament fabrication using an extruder with a single orifice die (Φ2 mm), (b) the EAM process (3D printing), (c) the welding workpiece, composed of two 3D-printed parts, and (d) the FSW process for the printed workpiece. As shown in Figure 1a, after PETG pellets were plasticized and extruded from the die, they were hot-stretched at an appropriate drawing ratio and the Φ1.75 mm filament was fabricated. The obtained filament was fed into the 3D printer and melted, as shown in Figure 1b, where the melt was pushed through the printer nozzle and then solidified as controlling in the three directions (X, Y, and Z), resulting in the production of 3D printing parts. To investigate the mechanical properties of the welding quality for 3D-printed samples, two 3D-printed parts were prepared separately, and their shapes and relative positions are shown in Figure 1c. The workpieces, which were composed of the two 3D-printed parts, were welded using the typical FSW process as shown in Figure 1d.

**Figure 2:**
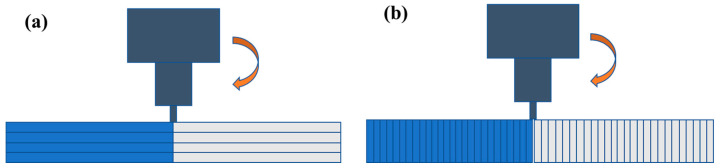
FSW processes for the parallel (**a**) and perpendicular (**b**) printed parts.

**Figure 3:**
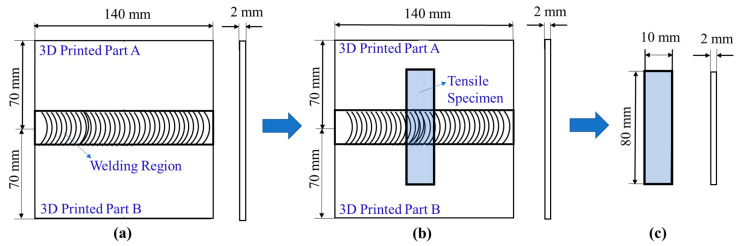
Shape and dimension of the welding workpiece (**a**), cutting position (**b**), and dimensions (**c**) of the tensile specimens.

**Figure 4:**
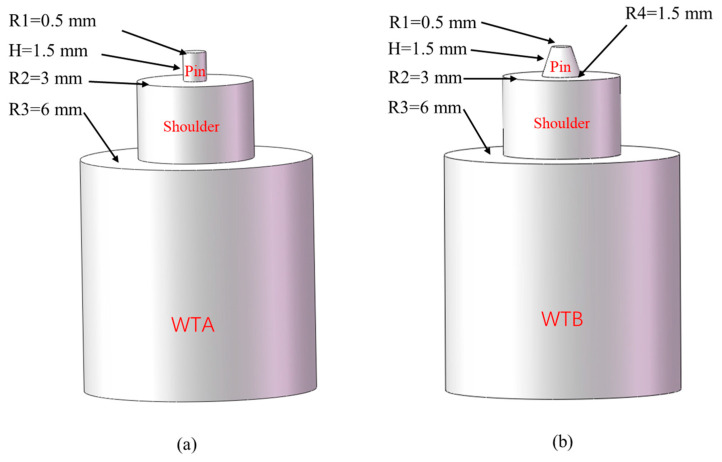
Specific parameters of the two different welding tools, (**a**) WTA and (**b**) WTB.

In order to investigate the effects of printing orientation, two methods were used to print parts, and their printing orientations were parallel and perpendicular to the *x* axis, which is the direction of the tensile test. For brevity, they were named parallel printing and perpendicular printing, and they are shown in Figure 2a,b, respectively. Before the experiment, the process parameters were optimized using orthogonal experimental methods [32,33]. The optimized printing process parameters are listed in Table 1. To facilitate subsequent welding, the sample size was determined to be 75 × 75 × 2 mm, and these dimensions are shown in Figure 3a. The welded samples were cut with a water-cutting machine, and samples each with a size of 80 × 10 × 2 mm were obtained for the tensile test. The cut position and the size of the specimens are shown in Figure 3b,c, respectively. Two types of welding tool, with a cylindrical shape (WTA) and a truncated conical shape (WTB), were designed and manufactured for welding experiments [34,35] separately, according to the PETG welding characterization requirements, and their specific shapes and dimensions are shown in Figure 4. For friction stir welding of polymer materials, the tool material is not required to be high, but a surface finish as high as Ra 0.1 is generally required. Optimized FSW process parameters are listed in Table 2.

### 2.3. Testing and Characterization

Following the standard set by GB-T/1040.1-2006 [36], the tensile test was carried out on a universal test machine (CMT4303) with a tensile speed of 5 mm/min at a room temperature of 25 °C. Five specimens from each group were selected for the test, and the average tensile strength and elongation at break were recorded for each specimen.

The morphologies of printed and welded samples were characterized by a scanning electron microscope (SEM) separately. The samples were cut from designated positions, which were generally perpendicular to the printing direction and welding direction, and then quenched in liquid nitrogen. Before observation, the fracture surfaces of the samples were sprayed with gold for 45 s to improve the quality observed. Furthermore, the tensile fractured surfaces of the welding workpieces were also observed after gold was sprayed directly. For the sample preparation method, one can refer to our previous research on injection molding samples [37,38,39,40,41,42].

## 3. Results and Discussion

### 3.1. Porosity and Anisotropy of 3D-Printed Parts

**Figure 5:**
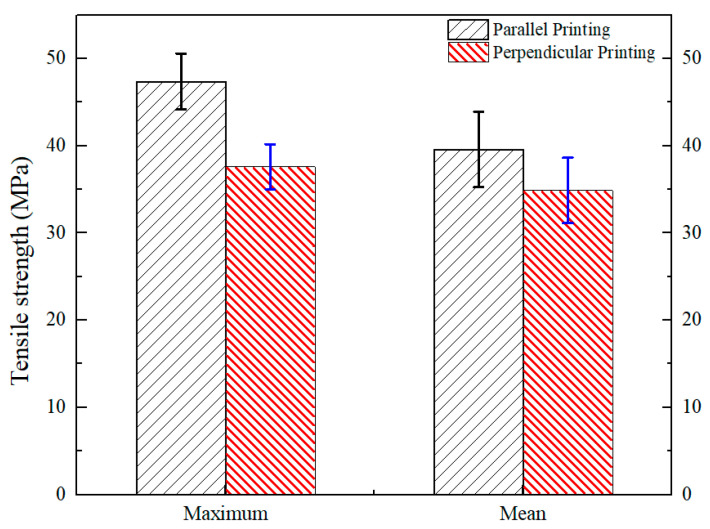
Comparison of the tensile strengths of parallel and perpendicular printing parts.

Typically, the mechanical properties of 3D-printed polymer parts are greatly affected by the direction of printing, as shown in Figure 5. The maximum and average values of tensile strength for parallel printing samples are 48.47 MPa and 39.59 MPa, which are 28.88% and 13.54% higher than those of perpendicular printing, respectively. The great difference in tensile strengths for different printing directions indicates that the anisotropy of the 3D printing PETG samples obviously exists. Actually, it is difficult to eliminate not only the anisotropy but also the inner pores of the printed parts only using the method of optimization of the processing parameters.

**Figure 6:**
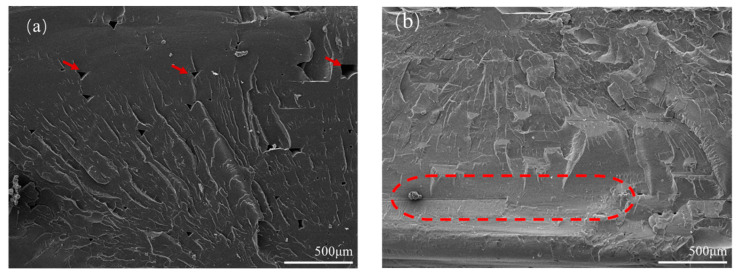
Inner structure of the EAM (**a**) parallel printing and (**b**) perpendicular printing sample using the optimized printing parameter.

**Figure 7:**
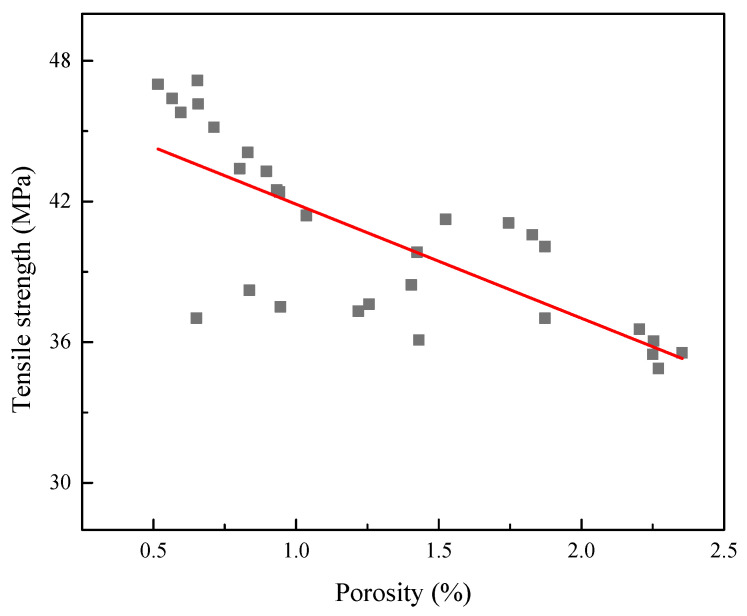
Relationship between tensile strength and porosity of 3D-printed parts.

The inner structures of the printing samples are characterized by SEM and the results are shown in Figure 6. It can be seen from Figure 6a that there are some obvious pores among the parallel deposited materials. Most pores have a diameter of about 50 uµm, and the maximum is even larger than 100 µm, as measured by image analysis software. As for perpendicular printing, as shown in Figure 6b, it seems that the pores do not exist. However, it can be known from a simple analysis of the image capture position that the gap between the deposited materials is located on the quenched cross-section, resulting in the inability to find pores in Figure 6b. After a series of tensile tests and morphological observations, the results have been represented as scattered data points in Figure 7. According to the results in Figure 7, it can be concluded that the porosity of the printed parts changes with the varying processing parameters in the EAM process, leading to great differences in tensile strength.

The relationship between porosity and tensile strength can be obtained by using a regression fitting with the least squares method, and the result is a line as shown in Figure 7. A linear relationship between porosity and tensile strength can be written as model (1) as follows.

*σ* = a + b × *p* (1)

where *σ* and *p* are tensile strength (MPa) and porosity, respectively, a and b are fitted parameters and represent the intercept of tensile strength and the slope of the fitting line, respectively, and *p* denotes porosity. Quantitative analysis of porosity based on the morphology obtained from the SEM images was performed using the image processing software package ImageJ^®^ V1.64R. The porosity used in this study is the plane ratio.

Using the least squares method, the values of a and b can be determined to be 47.745 and −4. 862 in this study, respectively, and the correlation coefficient *R*^2^ is 0.907, suggesting that tensile strength is negatively correlated with the porosity of the printed specimens. Therefore, it can be seen from model (1) that the tensile strength of the EAM parts increases with a decrease in porosity. It should be noted that the low correlation coefficient indicates that the factors that affect tensile strength are complex and further exploration is still needed.

### 3.2. Effect of Different Welding Tools on the Mechanical Properties of Printed Workpieces

**Figure 8:**
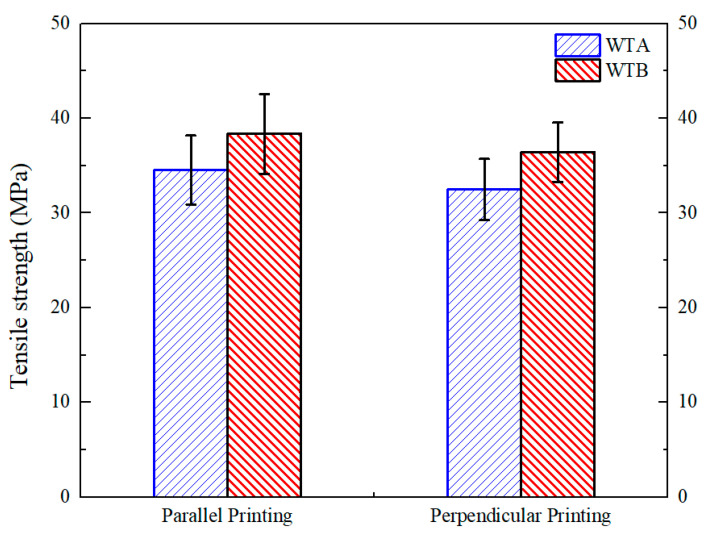
Mechanical properties of the welding workpieces using different welding tools.

**Figure 9:**
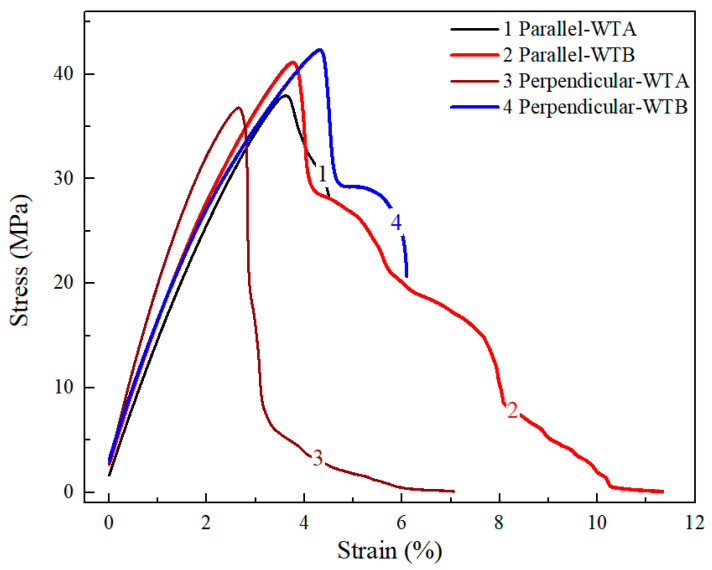
Representative stress–strain curves of welding workpieces using different welding tools.

It can be concluded from the above discussion that the EAM process is prone to defects in both anisotropy and high porosity. Therefore, FSW is used to reduce these two defects. The EAM parts in the two printing directions were welded using the two welding tools and their tensile strengths are shown in Figure 8. When WTA and WTB are used separately, the average tensile strengths of the parallel and perpendicular printed samples are reported to be 34.52 MPa and 32.47 MPa, and 38.36 MPa and 36.42 MPa, respectively. Increases of 11.12% and 12.16% for the tensile strengths of parallel and perpendicular printed specimens using WTB compared to those using WTA suggests that WTB in FSW provides more benefit to welding quality than WTA. To further compare the effects of different welding tools on mechanical properties, four representative stress–strain curves are shown in Figure 9. Overall, the tensile failure behavior of the four samples does not exhibit an immediate fracture after yielding, although the elongation-at-break is not high. For the perpendicular printed sample with WTA, the tensile strength suddenly decreases after the yield, but does not completely decrease to zero, indicating that a small portion of the material still maintains toughness, showing a ‘half-ductile’ character. Furthermore, the elongation-at-break of the welded parallel printing sample using WTB is 12.31%, and this value is higher than those of other samples, indicating that the use of WTB is beneficial to improve the mechanical properties of the welded workpieces.

The tensile properties of the welded samples can also be compared with the 3D-printed samples directly. It can be found that the tensile strengths of welded parallel-printing samples using both WTA and WTB are somewhat lower than those of the original parallel-printed samples. The low mechanical properties of the welded printing specimens are actually expected since the strength of the welded position is generally lower than that of the base materials. Instead, the tensile strengths of welded samples are almost equal to those of original perpendicular-printed ones. In particular, the tensile strength of the welded samples using WTB shows even an increase of 4.4% compared to the result of the original 3D-printed sample shown in Figure 5. The tensile fracture positions of the welded workpieces are generally not in the welding region, but rather, in the area of the base material. Therefore, it can be concluded that WTB is more conducive to improving the mechanical properties of welded perpendicular parts. Furthermore, compared to the anisotropy caused by the printing direction of the 3D-printed parts, the welding strengths of the workpieces fabricated by different printing directions show little difference, suggesting that the anisotropy of the printed specimens can be eliminated.

### 3.3. Mechanism of Effect of FSW on the Mechanical Behaviors of 3D-Printed Parts

**Figure 10:**
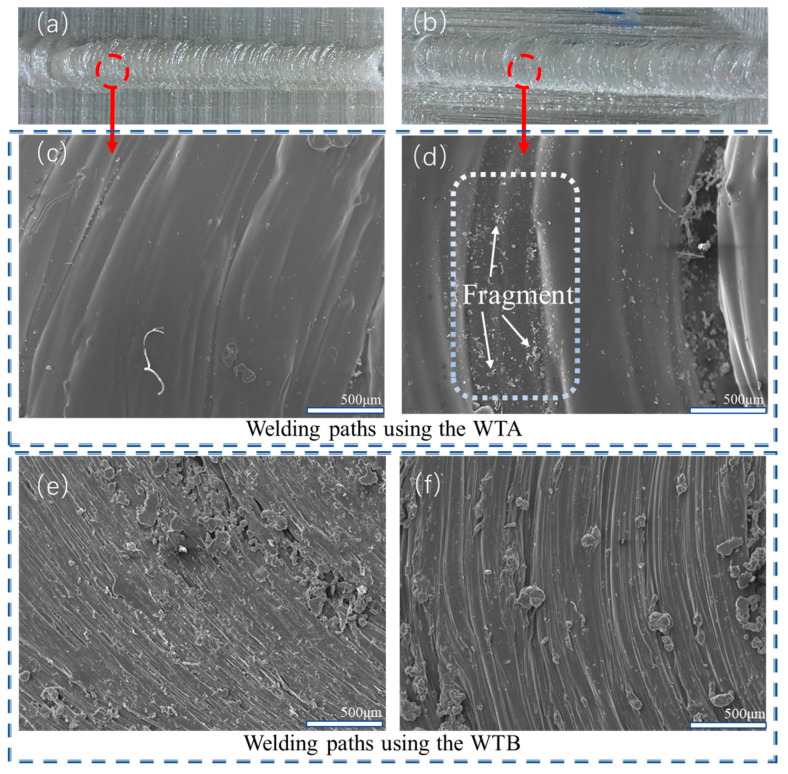
Surface diagrams of the welding regions for 3D-printed parts in (**a**,**c**,**e**) parallel and (**b**,**d**,**f**) perpendicular printing directions using WTA (**c**,**d**) and WTB (**e**,**f**).

**Figure 11:**
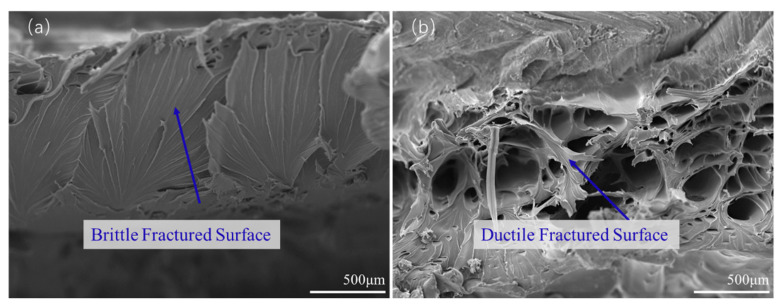
Tensile fractures of the welded parallel-printed parts with (**a**) WTA and (**b**) WTB.

**Figure 12:**
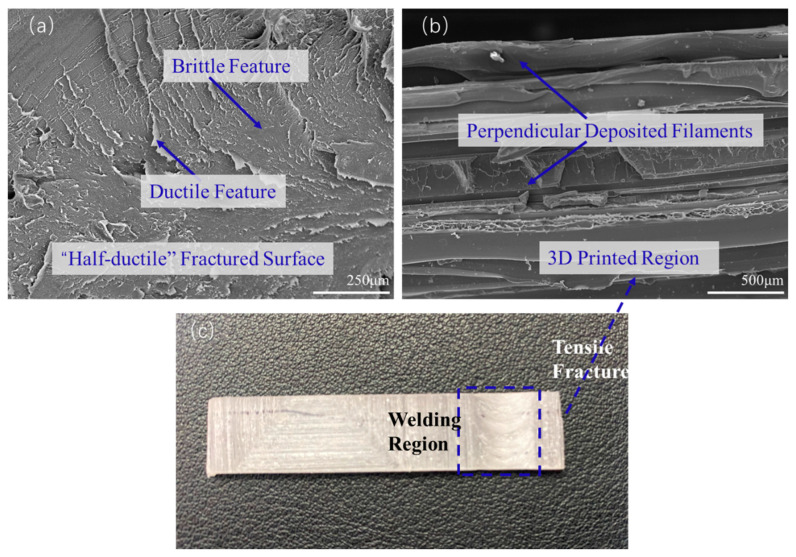
Tensile fracture of the welded perpendicular printing parts with (**a**) WTA and (**b**) WTB; (**c**) schematic diagram of the fracture behavior.

**Figure 13:**
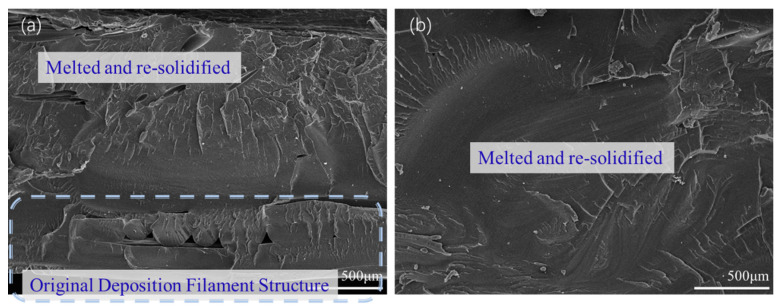
Quenching fracture surfaces of the welded parallel printing parts with (**a**) WTA and (**b**) WTB.

**Figure 14:**
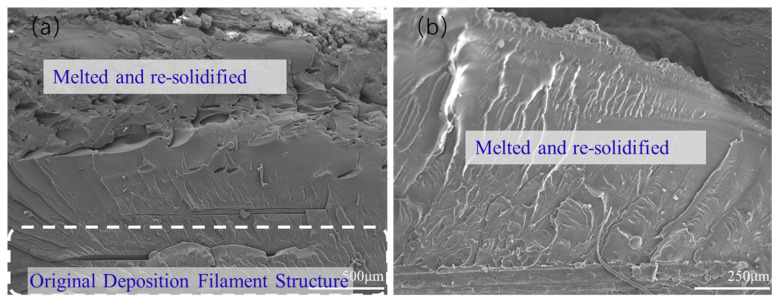
Quenching fracture surfaces of the welded perpendicular printed parts with (**a**) WTA and (**b**) WTB.

To further investigate the mechanism of FSW on the mechanical properties of the printed parts, the internal structures of different welded and printed samples were carefully characterized by using an optical microscope and SEM, and the results are shown in Figure 10, Figure 11, Figure 12, Figure 13 and Figure 14. Figure 10 shows the morphological results of the welded samples printed in parallel and perpendicular printing directions using both WTA and WTB. Figure 10a,b show the optical-microscope-observed surface of the welding regions of the parallel and perpendicular printing parts using WTA, respectively. There is not much of a difference between them, based on macroscopic observation, and the welding seams are both well formed in the welded region. However, slight differences between them can be found via SEM observation, and the results are shown in Figure 10c,d. Some fragments exist at the surface of the welded region, as shown in Figure 10d, and almost no fragment can be observed in Figure 10c. This phenomenon can be attributed to the printing directions of the welded parts. In the FSW process shown in Figure 10d, the welding direction is parallel to the perpendicular printing direction. The perpendicular deposited filaments in the welding region may rotate entirely together with the welding tool as a result of the function of the tangential force, resulting in the incomplete plasticization of some materials and some dispersed particles that have been formed. Compared to the WTA results, the welding paths of the parallel and perpendicular printing samples using WTB are more clear and fine, as shown in Figure 10e,f. There is almost no fragmentation on the welding surface, suggesting that the plasticization effect using WTB in the FSW process exceeds that using WTA. The advantages of WTB over WTA can be clarified from this point of view.

Figure 11 shows the tensile fracture surfaces of the welded workpieces of the parallel printing parts using WTA (a) and WTB (b). It can be seen from Figure 11a that large tear edges, caused by plastic deformation under the force in the tensile direction, appear on the surface when WTA is used. The flat cross-section indicates that the failure is brittle during tensile fracture, suggesting that the bonding of the material in the welding region is somewhat not enough. When the welding tool changes to type B, the failure mode obviously shows a ductile fracture, as shown in Figure 11b, which may be due to the better plasticization caused by the sufficient friction between the high-speed rotating tool and the materials.

The advantage of WTB over WTA with regards to the mechanical behavior of 3D perpendicular printing parts appears to be more evident, compared to that of parallel printing parts, as shown in Figure 12. When WTA is used, the fracture occurs in the welding region and the failure surface shows a ‘half-ductile’ fracture feature, as shown in Figure 12a. This means that there are also a few tear edges, indicating a fragile fracture, on the fracture surface, except for some ductile tearing places, as shown by the arrows in Figure 12a. This is also consistent with the results of the tensile test in Figure 9. When using WTB, some perpendicular deposited filaments can be observed on the surface of the tensile fracture, as shown in Figure 12b. The tensile fracture actually appears in the 3D-printed region rather than in the welding region, as shown in Figure 12c, indicating that the welding strength has exceeded the strengths of the perpendicular printed parts themselves. This is consistent with the previous comparison of tensile strengths, indicating that WTB can effectively influence the mechanical behaviors of the 3D-printed PETG parts.

The reason for our improving the mechanical properties of the welded workpieces using WTB can be attributed to the special shape of the tool. First, the conical surface of WTB is larger than the cylindrical surface area of WTA. During the welding process, the contact surface area between WTB and the based material is relatively larger, resulting in more heat being generated, which improves the flow of the material and the formation of a stronger welding joint. Furthermore, the irregular conical shape of WTB is more conducive to the mass transfer of materials than the regular cylindrical shape of WTA as it can promote the downward movement of the welding tool and reduce the welding resistance, leading to an improvement in the fusion effects of the welding region. Similar studies on the three-groove tapered threaded welding tool and the tapered threaded welding tool can be found in references [43,44,45,46]. Therefore, WTB is more beneficial to material flow, which can improve the mechanical properties of the welding region.

To further analyze the related mechanism of the effect of FSW on the mechanical behaviors of 3D-printed parts, the quenching fracture surfaces of the workpieces in the welding region that are parallel to the welding direction were also characterized by SEM for the parallel and perpendicular printing parts, and the results are shown in Figure 13 and Figure 14, respectively. In Figure 13a, there are obvious differences between the upper and lower parts. In the upper part of the workpiece, the pin of the welding tool can touch the materials directly and the PETG is obviously melted due to the friction heat resulting from the force of the pin and the shoulder of the welding tool. The molecular chains of PETG can be tightly entangled with the cooling of the melt, resulting in a tighter structure being formed. However, in the lower part of the workpiece, the pin cannot touch the materials directly, and the heat transferred from the upper part cannot melt the deposited materials thoroughly, resulting in an almost-unchanged deposited filament structure for the 3D printing parts. This is consistent with the previous discussion, indicating that the A-type design of the welding tool is not conducive to the welding of 3D-printed parts. This situation can be improved when using WTB, as shown in Figure 13b. Due to the special geometric structure of WTB, the touch between the pin of the tool and the based materials is more compact, and the heat transfer is more sufficient during the FSW process, which leads to a tight cross-section with almost no large pores, and even the original 3D-printed structure below the pin shows the features of melting and re-solidification. In addition, this suggests that WTB is beneficial for connecting the printing parts and improving the mechanical properties of the welding workpieces.

A similar situation can also be verified after the characterization of the welding region for perpendicular printing, as shown in Figure 14. In Figure 14a, WTA is used, the welding region where the tool is located is not touched, the perpendicular printing parts are not also completely plasticized, and the original structures of adjacent deposited filaments can still be observed, indicating that the degree of plasticization of the material is low during the FSW process. When using WTB, as shown in Figure 14b, the welding region is obviously tight and there are no large gaps or defects, suggesting high welding quality. Therefore, WTB has been proven to be more conducive to the welding of 3D-printed parts again, and this finding is attributed to the special geometric shape of WTB, which is more conducive to the flow of materials and the heat transfer during welding. Meanwhile, a larger surface area can generate more heat during welding, promoting entangled molecular chain formation and leading to a tighter welded joint being formed.

## 4. Conclusions

In this study, two types of welding tools were used to carry out the FSW process for PETG parts that were fabricated by an EAM process in two directions, parallel printing and perpendicular printing, and their mechanical properties and micromorphology were analyzed and characterized. It was found that 3D-printed PETG parts have obvious anisotropy, and the mechanical properties are obviously different because of the existence of internal pores. Therefore, a quantitative relationship between tensile strength and porosity was determined. The anisotropy was obviously reduced when the FSW process was completed for the 3D-printed parts. In particular, FSW using WTB was found to have a series of advantages with regards to the mechanical properties of 3D-printed parts. Further analysis of the potential mechanism suggested that WTB was more conducive to the welding of printed parts, which was attributed to the special geometric shape and the large surface area of WTB. Therefore, this study shows that hybrid manufacturing using the FSW and EAM processes is feasible and may be applied directly to modern industrial manufacturing. The study also provides a hint of improving the EAM process using high-speed fiction, and this method is on its way to further exploration.

## Figures and Tables

**Table 1:** Optimization of parameters of the 3D printing process.

Printing Direction	Parallel	Perpendicular
Melt temperature (°C)	260	260
Print speed (mm/s)	30	40
Hot bed temperature (°C)	90	90
Layer thickness (mm)	0.1	0.2

**Table 2:** Optimized process parameters of FSW.

	Parameter
Rotation speed (r/min)	3000
Welding speed (mm/min)	30
Press amount (mm)	0.2
Welding tool	WTA/WTB

## Data Availability

All data included in this study are available on request by contacting the corresponding author.
